# Active State Organization of Spontaneous Behavioral Patterns

**DOI:** 10.1038/s41598-017-18276-z

**Published:** 2018-01-18

**Authors:** C. Hillar, G. Onnis, D. Rhea, L. Tecott

**Affiliations:** University of California, San Francisco Department of Psychiatry, 1550 4th Street, San Francisco, CA 94158 USA

## Abstract

We report the development and validation of a principled analytical approach to reveal the manner in which diverse mouse home cage behaviors are organized. We define and automate detection of two mutually-exclusive low-dimensional spatiotemporal units of behavior: “Active” and “Inactive” States. Analyses of these features using a large multimodal 16-strain behavioral dataset provide a series of novel insights into how feeding, drinking, and movement behaviors are coordinately expressed in *Mus Musculus*. Moreover, we find that patterns of Active State expression are exquisitely sensitive to strain, and classical supervised machine learning incorporating these features provides 99% cross-validated accuracy in genotyping animals using behavioral data alone. Altogether, these findings advance understanding of the organization of spontaneous behavior and provide a high-throughput phenotyping strategy with wide applicability to behavioral neuroscience and animal models of disease.

## Introduction

A primary function of an organism’s central nervous system is the management of its relation to its environment through regulation of a complex set of interacting behavioral processes occurring over multiple spatial and temporal scales. However, the ways in which diverse behaviors are coordinately regulated are not typically addressed by focal assays addressing particular behavioral processes in isolation. Interpretation of results could thus be complicated by contextual factors, such as time-of-day, animal handling, test novelty, or influences of competing neurobehavioral processes that the assays are not designed to detect. Such considerations highlight a need to enhance the comprehensiveness of behavioral analyses so that experimental influences on particular endpoints may be interpreted in a broader behavioral context.

Toward this end, we have pursued a strategy inspired by Systems approaches for investigating the structure and dynamics of complex biological systems^1–3^. We have developed Home Cage Monitoring (HCM) methods that enable quantitative assessment of diverse behaviors spontaneously expressed by undisturbed animals in their home cages over short and long time periods. A benefit of this approach is the opportunity it provides to identify, in a hypothesis-independent manner, emergent properties of behavioral expression: higher-order features of behavioral organization that may not be detectable in studies focused narrowly on particular behavioral endpoints. Here we identify the “Active State” as such an emergent property with the potential to facilitate insights into the manner in which diverse behaviors are coordinately regulated in *Mus Musculus*^4–9^.

We had previously applied a multimodal analytical approach to HCM data, employing the Active State concept derived from field studies of small rodents. Mice and rats typically establish a nest or burrow in a sheltered location at which periods of relative inactivity occur. These “Inactive States” (ISs) are interspersed with “Active States” (ASs), periods during which animals emerge from these locations for active foraging excursions, during which feeding, drinking, and exploration occur^10–12^. Although this basic spatiotemporal pattern has been described in wild animals, it had not been explored as an organizing principle for quantitative behavioral analysis in laboratory animals. We had developed criteria for the automated classification and  quantification of HCM data into ASs and ISs and reported that AS/IS dynamics and home cage behaviors are sensitive to single gene energy balance mutations and pharmacogenetic manipulation of brain serotonin pathways^5,13^. Although the approach enabled detection of behavioral phenotypes with high sensitivity, it had been applied predominantly to mice with the C57BL/6 J genetic background. Therefore, its general applicability to studies of other strains and the extent to which AS/IS organization represents a fundamental organizational feature of behavior in *Mus Musculus* had been unclear.

We examined the generalizability of AS/IS spatiotemporal organization by studying the spontaneous behavioral patterns expressed by 16 genetically diverse inbred strains of mice^14^. A total of 170 mice were studied, and data were collected over 1921 “Mouse Days” (MDs; data collected for each mouse during each experiment day). We report that application of the AS/IS concept reveals a series of previously unknown features of behavioral organization and provides unprecedented accuracy in the behavioral phenotyping of mice.

## Results

### AS/IS organization of spontaneous behavioral patterns in *Mus Musculus*

**Figure 1:**
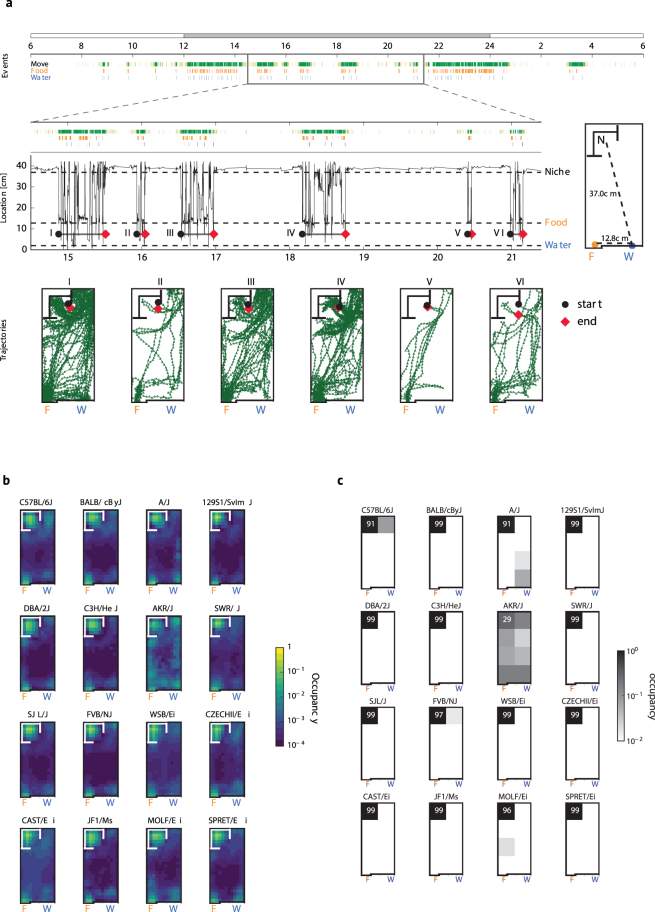
Spatial and temporal organization of mouse behavior. Figure 1a (top) displays a raster plot for a C57BL/6J mouse indicating temporal patterns of movement events within (yellow) and outside (green) the niche, as well as feeding (orange) and drinking (blue) events. Dark Cycle (Zeitgeber time (ZT) 12-24) and Light Cycle (ZT 0–12) are shown by gray and white rectangles above the rasters. Behavior occurring during the time period indicated by the rectangular box is displayed in further detail below (middle). Animal location, as assessed by distance from the water spout, is indicated on the Y axis. Distances from the water spout “W” to the locations of the Niche “N” center and Feeder “F” are shown on cage diagram. Six periods of activity (I-VI) were interspersed with long niche visits. Below this (bottom) are displayed movement paths corresponding to the activity periods, with their start and end locations indicated by the black circles and red symbols, respectively. Figure 1b displays average occupancy times across 24 h, with a 12 × 24 grid cage discretization. Occupancy values indicate the proportion of time spent within each cell. Values shown are strain averages over all MDs. Figure 1c displays average occupancy times during ISs, indicated with a 2 × 4 grid cage discretization. Strong preferences for the niche characterize all but the AKR/J strain.

We tested the hypothesis that the lives of caged laboratory mice exhibit features of an AS structure, such as the establishment of a single “Home Base”; i.e., a favored location at which long periods of inactivity (ISs) occur^12^. Characteristics of AS/IS organization are illustrated in Fig. 1a, which displays a representative record from a C57BL/6 J mouse. A highlighted portion of the nighttime record contains 6 periods of activity that were interposed with inactive periods. Examination of the movement paths expressed during these active periods revealed that each began with an excursion from the nesting niche and that each terminated with a return to the niche.

To determine how animals spatially allocate their time, we generated position density plots for each of the 16 strains. These revealed highest occupancy times in the niche, with secondary peaks at the feeder (Fig. 1b). For each MD, we designated a Home Base area by spatially discretizing the cage area into a 2 × 4 array of cells (one of which contained the entire niche area) and calculating occupancy times. Home Bases were designated as the cells with peak occupancies, and for all MDs, these corresponded to the nest location determined by daily visual inspection.

To formally define ASs, we collected the union of movement, feeding, and drinking events while animals were outside the Home Base and connected temporal gaps between events of lengths less than a threshold duration value (IST; “Inactive State Threshold”). The resulting collection of time intervals were designated as ASs (Fig. S1). ISs were then defined as the complement set of these ASs. We assessed the robustness of this AS/IS designation method by examining the impact of a wide range of IS thresholds on AS Numbers. AS designation was robust for all strains, as indicated by marked similarities in AS Numbers using IS thresholds ranging from 15 to 30 min (Fig. S2). Moreover, high heritability of AS properties, as well as optimal cross-validated discrimination of mice by strain were achieved using a 20 min IST, a value used for all subsequent analyses.

We examined the distribution of spatial occupancies during ISs for all animals. Excluding the AKR/J mice, 156/158 animals exhibited much higher IS occupancies at the niche than elsewhere in the cage (Fig. 1c). The AKR/J group was an exception, as only 2/12 of these mice established nests in the niche. Nevertheless, the behavioral patterns of individual AKR/J mice displayed on each MD a robust AS/IS organization: each animal established a single nest at a single Home Base location at which long IS pauses occurred.

### Daily patterns of AS expression are strain-specific and correlate highly with movement and ingestion

**Figure 2:**
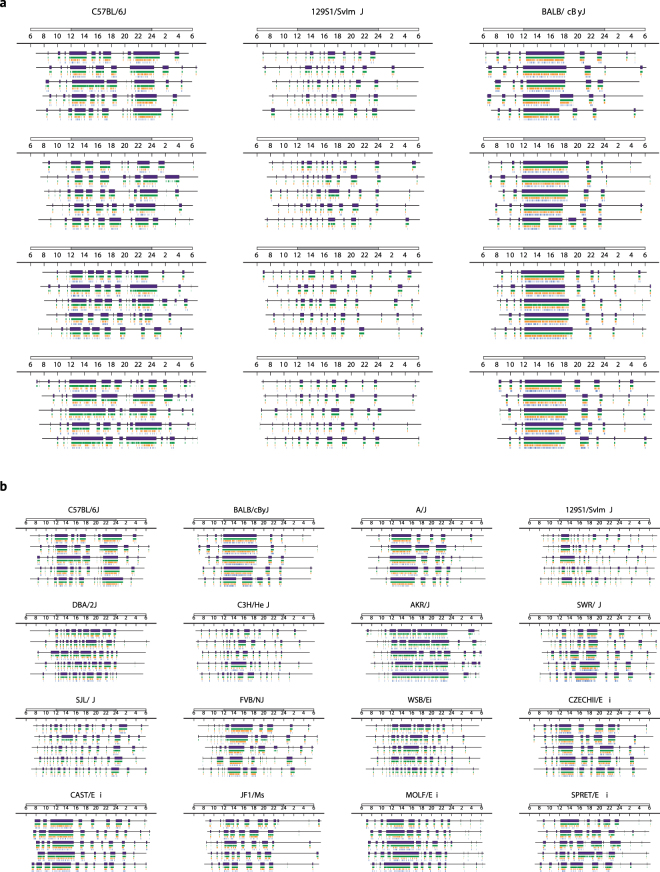
Strain-specific temporal patterns of AS expression. Figure 2a displays raster plots corresponding to 5 days of monitoring for 4 representative mice from each of three strains (C57BL/6 J, 129S1/SvImJ, BALB/cByJ) indicating temporal patterns of movement (green), feeding (orange), and drinking (blue) events. Purple bars indicate Active States. Dark Cycle (ZT 12–24) and Light Cycle (ZT 0–12) are shown by gray and white rectangles above the rasters. Representative individuals were those animals whose 2 h time bin AS Probability vectors were nearest their strain averages. Figure 2b displays raster plots for all strains. The representative individual for each strain was the animal whose 2 h time bin AS Probability 11-D vectors was nearest its strain average.

For all strains, the numbers and durations of ASs varied markedly with time-of-day. As expected, AS Numbers and Durations were generally greater during the nighttime than during the daytime. Typically, during the night, long duration ASs were interspersed with ISs, while during the day, long ISs were interspersed with briefer ASs. Examination of raster plots from individual mice depicting ASs and the behavioral events occurring within them revealed stability in daily patterns of ASs across consecutive MDs (Fig. 2a). Moreover, similarities in the daily patterns of ASs were observed among mice within each inbred strain group. By contrast, inspection of raster plots revealed substantial between-strain differences in daily patterns of AS expression (Fig. 2a,b). Heritability estimates for averaged (across 24 h) AS Probability and Distance Traveled were 0.72 and 0.49, respectively. Heritability estimates for each of the 11 2 h time bins were also calculated, and values for AS Probability consistently exceeded those for Distance Traveled (Table 1).

**Table 1:** Broad-sense heritability estimates *H*^2^ (mean, sd over 20 bootstrapped trials using random halves of the MDs) for AS Probability and Distance Traveled measures: 11 2 h time bins (Zeitgeber times) and their daily averages.

	8–10	10–12	12–14	14–16	16–18	18–20	20–22	22–24	0–2	2–4	4–6	Daily
ASP	0.77	0.84	0.86	0.56	0.68	0.49	0.76	0.64	0.72	0.40	0.44	0.72
sd	0.02	0.01	0.01	0.02	0.02	0.02	0.02	0.01	0.02	0.03	0.04	0.01
Dist	0.53	0.64	0.54	0.29	0.38	0.24	0.39	0.54	0.61	0.27	0.18	0.49
sd	0.02	0.02	0.01	0.03	0.07	0.06	0.04	0.03	0.02	0.04	0.04	0.02

**Figure 3:**
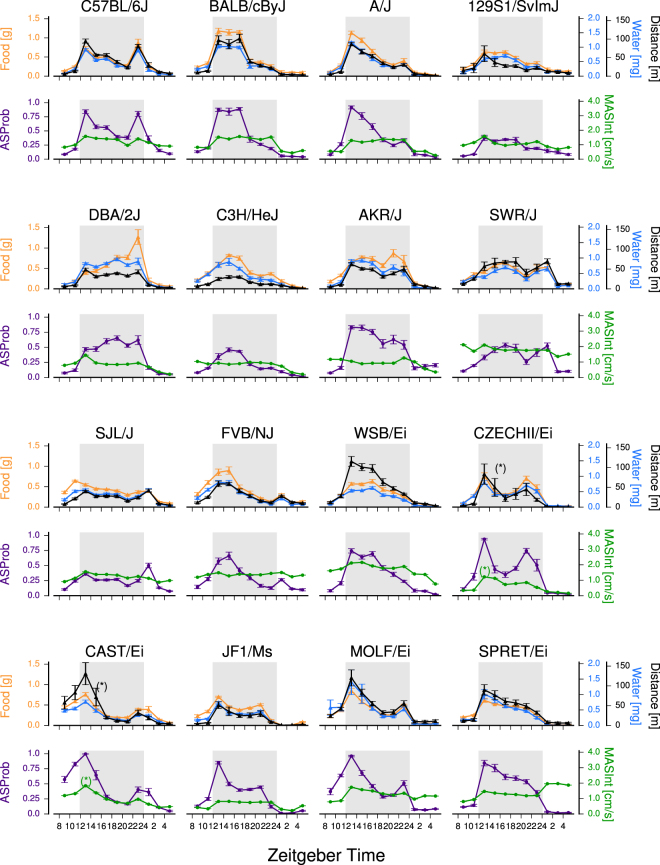
Daily patterns of ingestion, Distance Traveled, and AS properties. Plots of daily patterns (mean +/− sem) of Distance Traveled (black), food (orange), and water ingestion (blue) are shown in the top row for each strain set. These patterns resembled those of AS Probability (purple), but not AS movement Intensity (MASInt; green) displayed in the bottom row for each strain set. Distance Traveled and AS movement Intensity Y axes for strains CZECH and CAST were rescaled by 0.25 (*) due to their hyperlocomotor phenotypes.

Food intake, water intake, and Distance Traveled also varied markedly with time-of-day for all strains (Fig. 3). Within each strain, these behaviors varied in a similar manner, with extremely high correlations observed among them across the 24 h day (Fig. S3). We sought to determine whether these daily patterns were associated with fluctuations in the likelihood of expressing ASs at particular times of day (AS Probability) vs. fluctuations in the intensities of behaviors expressed during ASs (AS Intensities; i.e. amounts consumed or Distance Traveled per min AS time). Whereas daily patterns of AS Probability correlated extremely well with feeding, drinking, and Distance Traveled, daily patterns of the corresponding AS Intensities did not (Figs 3, S3).

### Insensitivity of AS time allocations to the light/dark cycle

**Figure 4:**
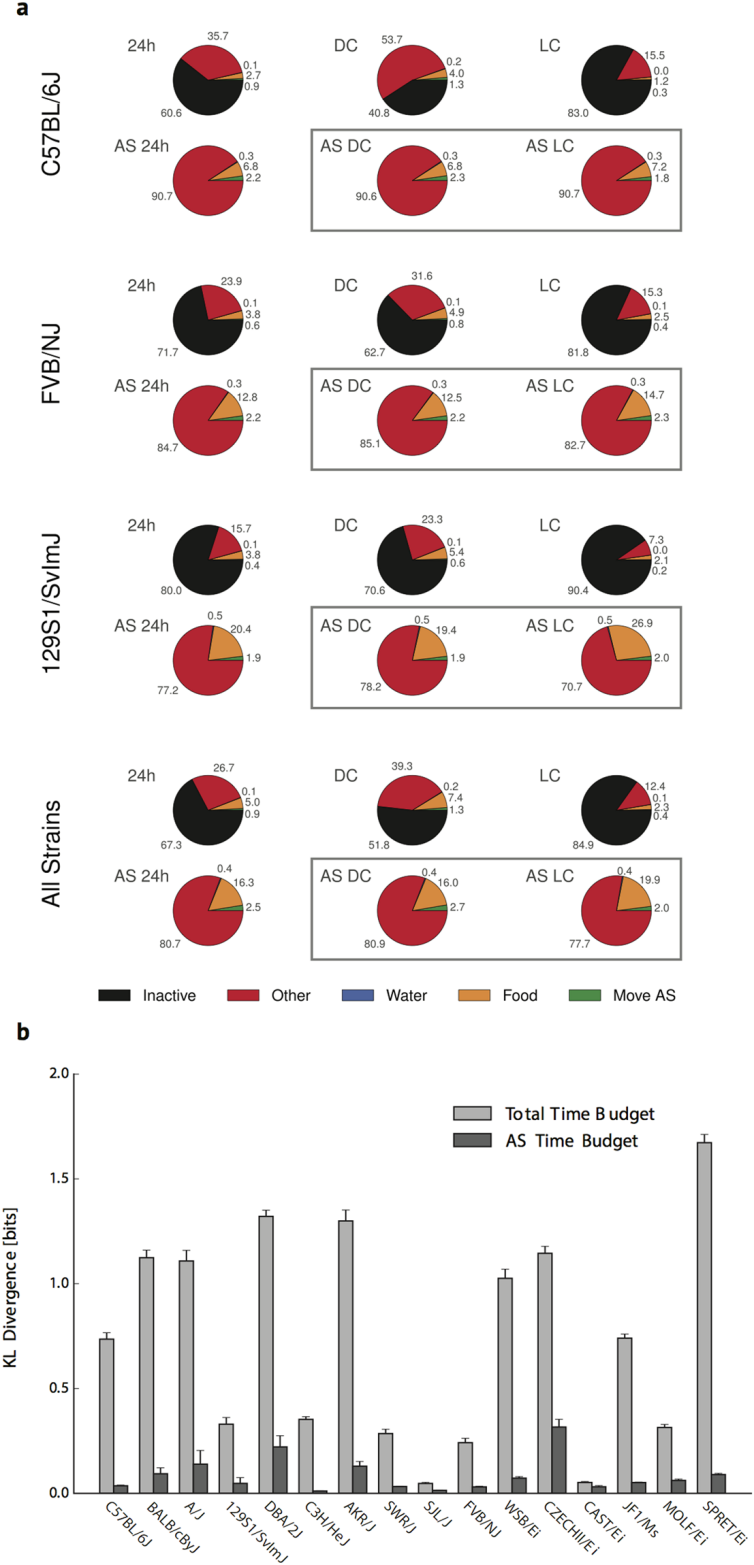
Time budgets. Figure 4a displays strain average time budget values for the C57BL/6 J, FVB/NJ, and 129S1/SvImJ strains. For each strain, the top row displays averaged Total time budgets corresponding to 24 h (24 h), dark cycle (DC), and light cycle (LC). Bottom row displays averaged AS time budgets corresponding to 24 h (AS 24 h), dark cycle (AS DC), and light cycle (AS LC). Below this are displayed time budgets derived from averaging values for all strains in the study. Figure 4b displays Kullback-Leibler divergence scores that indicate the extent to which each strain’s DC time budget can be discriminated from its LC time budget. Strain averages and standard deviations across 20 bootstrap trials (using random halves of the total data as input) are shown.

Application of the AS/IS concept revealed additional aspects of behavioral regulation exhibiting differential sensitivity to time-of-day. “Total time budgets” were generated to assess the allocation of time among various behaviors (IS and within-AS behaviors). Examples for the strains C57BL/6 J, FVB/NJ, and 129S1/SvImJ are shown in Fig. 4a. Breakdown of time budgets separately for the 12 h light period (“day”) and 12 h dark period (“night”) revealed, as expected for a nocturnal species, increased IS time allocations during the day, relative to the night.

We also examined time-of-day influences on the allocations of time to behaviors occurring *within* ASs by generating “AS time budgets” that excluded time spent in ISs. In contrast to the marked day-night differences observed in total time budgets, time allocations within nighttime ASs were very similar to those occurring during daytime ASs for individual strains and for data combined from all mice in the study (Fig. 4a). The extent to which this pattern generalized across all strains was evident using Kullback-Leibler divergence (Fig. 4b), a measure of the distance between dark and light period time budget distributions. Time-of-day influences on AS Probability and within-AS time allocations are thus dissociable: whereas the former vary markedly with time-of-day, the allocations of time among behaviors occurring within ASs are relatively stable throughout the 24 h day.

### AS onsets and offsets are associated with food and fluid intake

**Figure 5:**
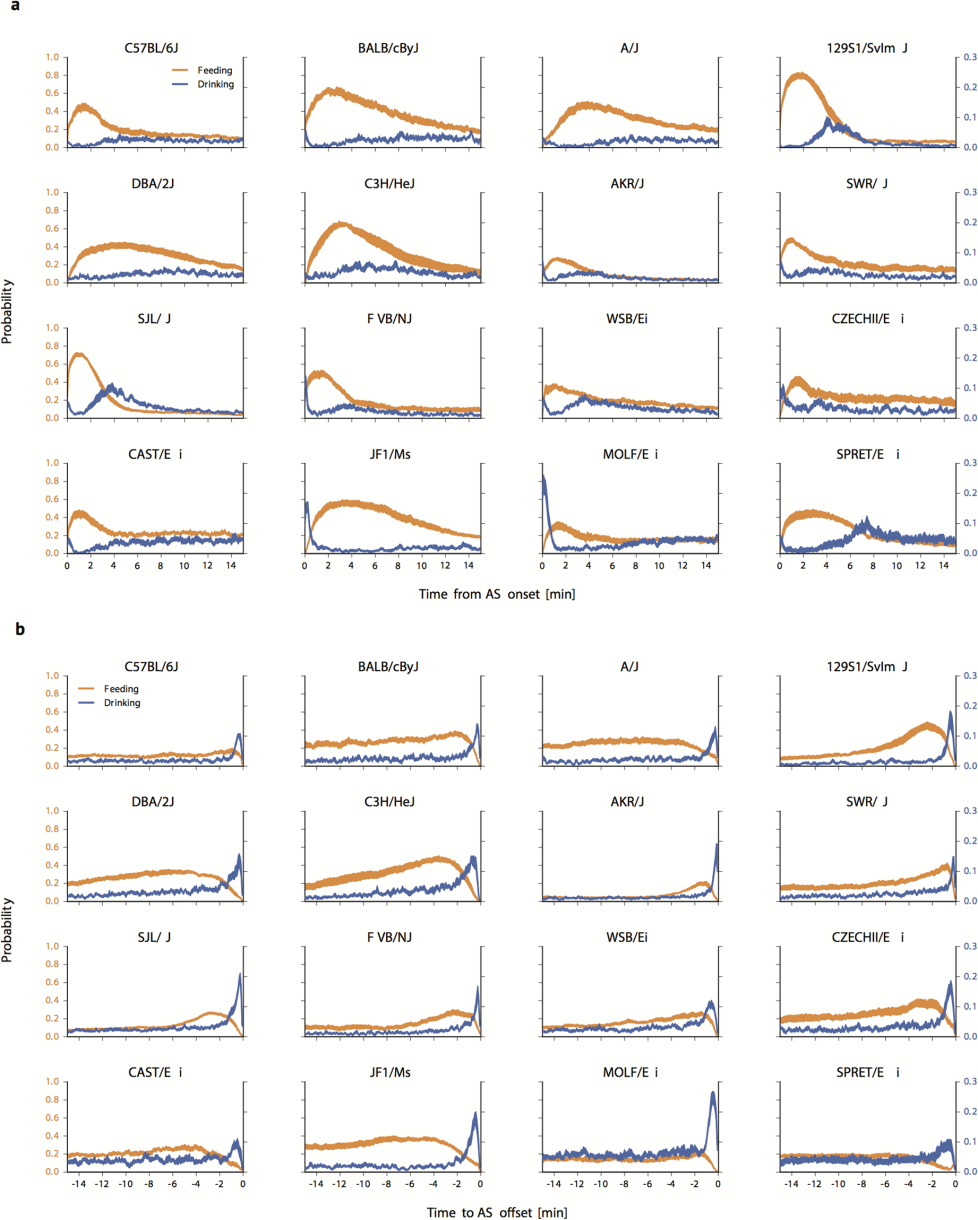
AS temporal structure. Figure 5a displays probabilities of feeding (orange) and drinking (blue) during the 15 min period following AS initiation (mean +/− sem). All feeding peaks at onsets had p-values < 0.001. Figure 5b displays probabilities of feeding (orange) and drinking (blue) during the 15 min period preceding AS termination (mean +/− sem). All drinking peaks at offsets had p-values < 0.001, except for peak drinking before AS offset in SPRET/Ei (p-value < 0.01).

We sought to determine whether a temporal organization of behaviors could be detected *within* ASs. We aligned AS onsets and determined how the probability of engaging in feeding and drinking behaviors varied with time from onset. A pattern was apparent for all strains (Fig. 5a), with feeding probabilities highest early in the AS, followed by a decline that varied among strains. Drinking probabilities were more variable early in ASs, with some strains exhibiting peaks as feeding probabilities declined, and others exhibiting peaks that preceded peaks in feeding probability. These patterns were found in ASs regardless of whether they occurred during the light or dark cycles. To detect behavioral patterns associated with AS termination, we aligned AS offsets. An end-of-AS pattern was also observed for all strains (Fig. 5b), with drinking probabilities peaking within 1 min of AS termination. These findings raise the possibility that the regulation of AS onsets and offsets may be linked to the regulation of energy and fluid balance in mice.

### Near-perfect strain classification from behavioral measures

We developed a machine learning approach to classify animals by strain. We first determined whether particular behavioral features discriminate MDs from pairs of strains by performing a clustering analysis on 2 h time bin quantities of either AS Probability or Distance Traveled. Half of each animal’s MDs were randomly assigned as a “Train” set, with the remainder serving as a “Test” set for cross-validation. Using the Train dataset as input to the unsupervised *K*-means algorithm (*K* = 2), we obtained two 11-D Train “centroids” for each pair of strains, representing the average 11-D vector of MDs in each pairwise discrimination. We then used these Train centroids to determine “Clustering accuracy scores” ranging from 0.5 to 1, with random performance indicated by 0.5 and perfect separation by 1. Test set and Train set accuracy scores for each of the 120 possible pair-wise comparisons of 16 strains were extremely similar, indicating high levels of robustness and generalizability. To estimate the sensitivity of clustering, we used a bootstrapping approach, repeating the entire analysis 20 times. We found that AS Probability and Distance Traveled data provided average accuracy scores of 0.91 and 0.82, respectively, for the 120 pair-wise comparisons (Fig. S4).

**Figure 6:**
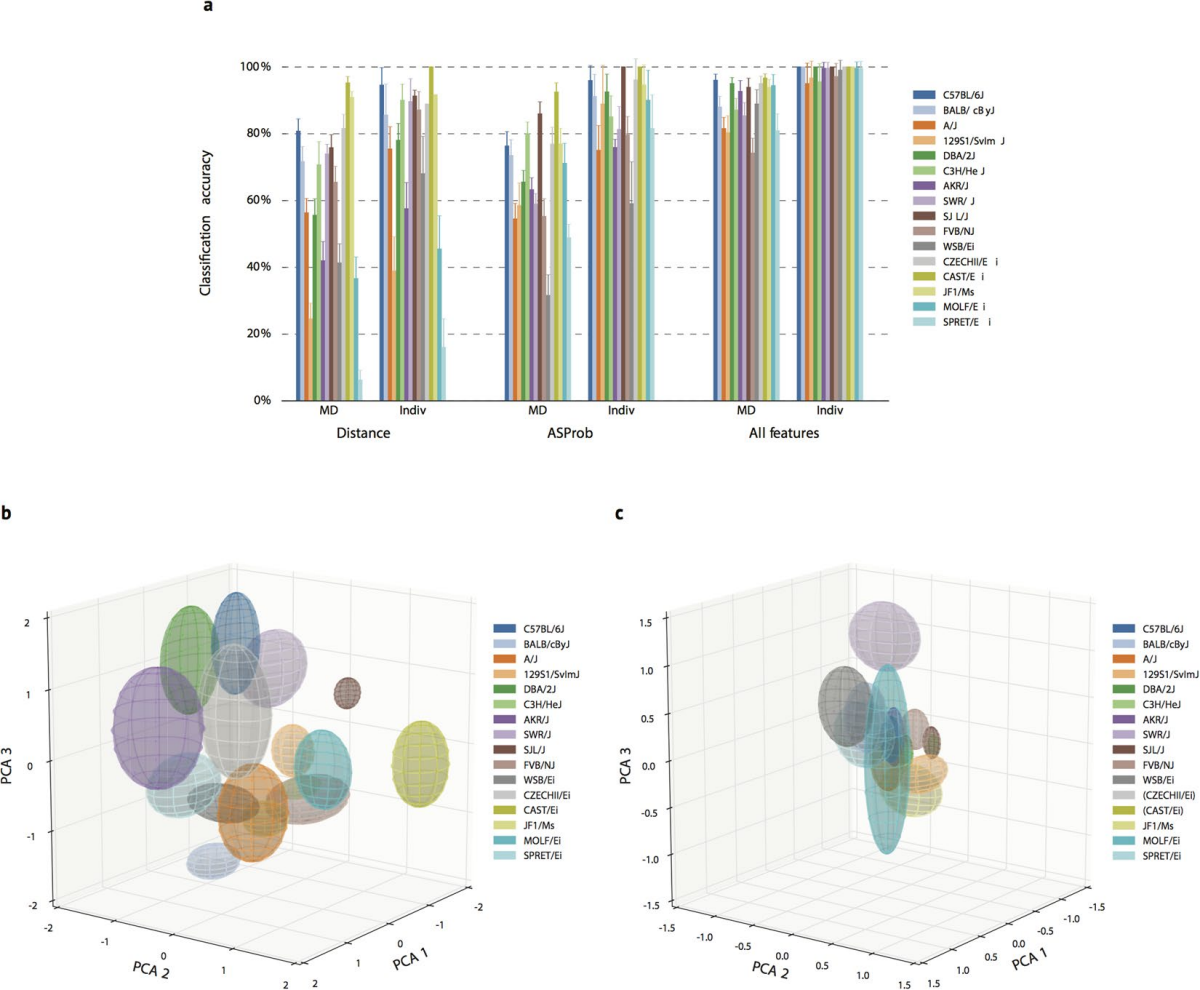
Segregation and classification of strain HCM datasets. Figure 6a displays output from a Full Strain Classifier. Classification accuracies for both single MDs (1921 total) and for averaged MD values from each mouse (170 Individuals) are shown. Cross-validated classification accuracies were derived from Distance Traveled, AS Probability, or combined HCM features determined over 20 bootstrapped trials (mean +/− sd), each with a random half of the data chosen as a Train set and the remaining MDs excluded as a validation Test set. Figure 6b displays three-dimensional projections of AS Probability data from each strain. Axes correspond to the top 3 normalized principal components of the 170 11-D average AS Probability mouse feature vectors. Each strain is represented by an ellipsoid with strain average at the center and with standard deviation in each component direction as the length of semi-principal axes. Figure 6c displays three-dimensional projections of Total Distance data from each strain. Axes correspond to the top 3 normalized principal components of the 170 11-D average Total Distance mouse feature vectors.

We next implemented a “Full Strain Classifier” that used regularized logistic regression to label individual MDs and individual mice into one of the 16 strains (for each mouse, chance is 6.25%). The resulting cross-validated strain classification accuracies are shown in Fig. 6a. AS Probability data yielded better classification accuracies (87%) than did Distance Traveled data (75%). Remarkably, the combination of AS and standard HCM features (see Methods) provided 99% classification accuracies for individual mice and 89% accuracies for individual MDs (Fig. 6a). We repeated the above procedure using 10% (instead of half) of the MD data (AS and standard HCM features) as a Train set, and found that even this relatively small Train set enabled high levels of classification accuracy (93% for individual mice and 76% for individual MDs).

To visualize how AS Probability alone segregates strain data, we performed a Principal Components Analysis (PCA) on the AS Probability feature vectors from all 170 mice (Fig. S5). This enabled projection of the data into the 3 dimensions corresponding to principal components with the most feature variance, revealing clear segregation of the strain data into 16 regions corresponding to each of the 16 strains (Fig. 6b). Visual discriminability achieved using AS Probabilities was higher than that using Distance Traveled data (Fig. 6c), and this was quantitatively confirmed by estimating the extent of overlap in all 120 possible pairwise combinations of the 16 regions (Fig. S6).

## Discussion

Transformative advances in neuroscience are being accelerated by Systems approaches characterized by the analysis of large datasets to elucidate the structure and dynamics of complex biological systems^15–17^. Although such strategies have been most frequently applied to molecular level datasets, their emphasis on comprehensive data acquisition and analyses over multiple spatial and temporal scales may also be productively applied to mammalian behavior. Toward this end, we have developed an approach involving the collection and analysis of high-resolution datasets that reflect the wide range of behaviors spontaneously expressed by mice in their home cages. Here we describe its application to a large dataset derived from a genetically diverse collection of 16 inbred mouse strains. From this dataset, we extract and validate an emergent organizational feature of behavioral expression in *Mus Musculus:* Active and Inactive States. We report the utility of these behavioral metrics for: (1) revealing novel insights into the manner in which animals coordinately express diverse elements of their behavioral repertoire and (2) a machine learning approach enabling unprecedented levels of precision in rodent behavioral phenotyping.

The assessment of home cage behaviors has been previously used to complement common focal behavioral assays. Although focal assays are essential for exploring a number of behavioral domains, they are also prone to limitations, such as their focus on a narrow range of behavioral endpoints, experimental variability produced by animal handling, labor requirements, practicality for examining time-of-day effects, and confounding test-order effects that can occur in animals subjected to multiple testing procedures^18–20^. Many of these concerns are obviated by the capacity to automate the collection of data that reflect the multiple behaviors spontaneously exhibited by undisturbed animals in their home cages. The ability to examine the simultaneous expression of multiple behaviors over short and long time scales enables development of a comprehensive approach for elucidating the manner in which behavioral patterns emerge from the coordinated regulation of physiological, affective, and cognitive processes.

Typically, studies of home cage behavior focus on the collection of standard behavioral endpoints, binned by time-of-day, and we found that strain values for body weights and gross behavioral measures such as food intake, water intake and Distance Traveled levels were in strong accord with published values (Jackson Laboratory Mouse Phenome Database: https://phenome.jax.org)^7,21,22^. However, there are at least three aspects of this work that distinguish it from other approaches. First, a primary purpose was to determine the extent to which genetically diverse inbred mouse strains exhibit common features of behavioral organization, particularly AS/IS organization. Behavioral (and most other) characteristics of inbred strains are known to vary widely, but those that generalize broadly across strains are more likely representative of the *Mus Musculus* species. Second, we used our HCM strain dataset to demonstrate that parsing behavioral records into Active and Inactive States enabled detection of previously unrecognized organizational features of behavior that generalized widely across strains. Third, we are unaware of prior reports that demonstrate levels of behavioral phenotyping precision sufficient to achieve the 99% classification accuracy reported here.

A basic invariant of spatial organization displayed by all strains was the establishment of a Home Base at which animals nest and exhibit their longest periods of inactivity. Moreover, each animal established a *single* Home Base location: multiple Home Bases were not observed for any strain. Animals strongly preferred establishing their nests in the niche enclosure, a finding consistent with field observations that mice favor nest locations with dense ground cover or within burrows^12^.

The key criterion for AS classification is the IS Threshold: the pause duration above which a subsequent behavioral event is considered to initiate a new AS rather than a within-AS pause. We find that over a wide range of IS Threshold values, there was little change in AS numbers, along with consistently high interstrain classification accuracies. We demonstrate that the lives of laboratory mice can be robustly parsed into mutually-exclusive ASs/ISs and that the application of AS criteria reveals a series of organizational features of behavior broadly characteristic of the Mus Musculus species:• Behavior can be robustly parsed into mutually exclusive ASs and ISs.• ISs are restricted to a single nest location.• Daily patterns of ASs are stable across days.• Daily patterns of ingestion and movement are highly correlated with AS Probabilities, but not AS Intensities.• Whereas AS Probability is sensitive to time-of-day, time allocations among behaviors occurring within ASs are not.• Probabilities of feeding peak near AS onset.• Probabilities of drinking peak near AS offset.• Daily patterns of AS Probability are exquisitely strain-specific.

We furthermore discovered that examination of AS/IS properties revealed patterns reflecting ways in which the expression of movement, feeding, and drinking were coordinately expressed throughout the day. Daily rhythms of these behaviors were highly correlated with daily rhythms of AS Probabilities. By contrast, daily rhythms of AS feeding, drinking, and movement Intensities were not correlated with temporal patterns in the expression of these behaviors. These findings raise the possibility that neural processes regulating daily patterns of AS Probability may contribute to the daily rhythms observed in multiple behavioral measures. Moreover, they indicate that distinct properties of ASs–AS Probability and AS Intensities–are dissociable and differentially sensitive to time-of-day.

The time budget findings also revealed previously unrecognized features of behavioral expression that are differentially sensitive to time-of-day. In accord with the known nocturnal pattern of mouse behavior, Total time budgets revealed substantial day-night differences, characterized by increased IS time during the 12 h light period, consistent with the marked time-of-day influence on AS Probability discussed above. By contrast, we found that time allocations among behaviors occurring *within* ASs (assessed by AS time budgets) were relatively insensitive to time-of-day. This phenomenon generalized broadly among all tested strains.

The utility of the AS/IS concept for revealing basic features of behavioral organization is further highlighted by examination of the temporal organization of behaviors occurring within ASs. Specifically, we observed predispositions of animals to feed at the onset of ASs and to drink immediately prior to AS offsets. These features of behavioral organization also generalized across all strains, and may thus represent inherent features of behavioral regulation in *Mus Musculus*. These findings enable generation of testable hypotheses regarding potential functional relationships between AS/IS transitions and homeostatic mechanisms underlying energy and fluid balance.

The observation that feeding occurs soon after AS onsets is reminiscent of what has been termed the basic rest activity cycle (BRAC)^23^, a recurring temporal pattern of autonomic nervous systems measures and behavioral events initially found in rats. The model reported by Blessing and colleagues describes a cycle commencing with sympathetic autonomic nervous system activation followed by behavioral activation and feeding behavior at the start of active periods. Although the impact of time-of-day on BRAC properties are not clear, such work nevertheless indicates that studies examining the coordinated regulation of AS expression and autonomic nervous system activity warrant consideration.

In addition to facilitating insights into the organization of behavior, the AS concept also enables a remarkable degree of phenotyping precision. To achieve this, we applied a modern practice in the analysis of large biological datasets: quantifying the degree to which differently labeled subgroups can be discriminated by a machine learning algorithm, with as little specification of ground truth labels, or “supervision”, as possible. This approach revealed that temporal regulation of AS properties were exquisitely sensitive to strain, as indicated by cross-validated accuracy levels of 99% for individual mice and 89% for individual MDs. As the dataset consists of 84.1 million behavioral events from which nearly 24,000 ASs are derived (a 3600 to 1 data reduction), the heavily coarse-grained AS/IS approach provides remarkable power across multiple analysis domains. Additionally, as was the case for heritability analyses, AS Probability outperformed a standard measure (Distance Traveled) for classification.

The classification accuracies reported above attest not only to the utility of AS analysis, but also to the quality of HCM data. The fact that they were achieved using data collected from 7 experimental runs (the 7 separate cohorts typically included 1-2 mice per strain) conducted over an 11 month period further indicates the reliability and replicability of HCM data. The relatively low (for rodent behavioral studies) levels of observed variability are likely the result of several factors, including: 1) absence of handling-induced behavioral disruption, 2) high spatial and temporal resolution of HCM measurements, 3) the inclusion of a habituation period to enhance subsequent stationarity of data, 4) standardization of the test environment (the HCM cage), and 5) the collection of 12 days of data per mouse. Although we collected data over an extended period of time, the quality of the data indicate that substantial precision could be obtained with experimental durations brief enough to warrant use of this approach for high- throughput screening applications. This point is highlighted by our finding that 93% classification accuracy for individual mice was achievable using just 10% of the data collected in this study. The feasibility of home cage data collection for high throughput screening purposes has already been demonstrated in circadian rhythm studies^24,25^. Our findings indicate that HCM testing can be suitable for an even broader range of high-throughput applications relevant to energy balance, volume regulation, physical activity levels, and their interrelationships.

The classification accuracies obtainable using limited portions of the 16 strain dataset also have implications for machine learning applications in the biological sciences. In the vast majority of instances, at least 80% of collected data are required as a Training set to achieve useful classification accuracies for the remaining data^26^. By contrast, the classification accuracies achieved here using just 10% or 50% of our MD data for Training (93% and 99% respectively) indicate that this dataset could provide a useful tool for those seeking to develop statistical and machine learning approaches to the study of behavior. Several benchmark mammalian datasets are currently in use for the development of machine learning applications, but to our knowledge none are derived from data with the high levels of volume and depth found here. Altogether, the above considerations indicate that this dataset, in combination with effective projections onto basic behavioral units (ASs/ISs), may be generally useful for the development of novel machine learning applications in the biological sciences.

We anticipate that the utility of the HCM approach will be further enhanced in the near future by the inclusion of sensors and hardware that will expand the volume and diversity of information that may be obtained from caged animals. One such limitation of the current system is its inability to distinguish the variety of nonlocomotor movements currently grouped within the “Other” category of our time budget analyses (eg: rearing, sniffing, digging, grooming). This will be addressed in a subsequent iteration of our system in which activity platforms are replaced by video monitoring capabilities that have been shown effective for discriminating among these behaviors and for the tracking of multiple animals in an enclosure^8,27,28^. Depending on the focus of study, one can readily envision the incorporation of sensors enabling acquisition of a wide variety of additional data streams (eg: wheel-running, autonomic measures, EEG, calorimetry, etc.).

At a more conceptual level, this work represents an experimental strategy that combines principles of Systems Biology with principles inspired by ethology: a focus on the objective definition and comprehensive analysis of the diverse behaviors that comprise a species’ behavioral repertoire. The identification of the AS as an emergent fundamental feature of behavioral organization provides an initial step for addressing an important challenge: development of a principled description of behavioral elements exhibiting structured patterns that can account for complex behavioral phenomena. A next step toward the development of a vocabulary for describing behavioral organization is the identification of behavioral elements that occur *within* ASs. For example, ingestive behaviors and locomotion do not occur continuously during ASs, but are instead clustered into bouts. Procedures for identifying and automating the detection of these bouts, and in turn the brief behavioral events from which they are composed, will provide the groundwork for a hierarchical model of behavioral organization that could usefully inform inquiry into its neural bases. The comprehensiveness and sensitivity of this approach may be particularly useful for explaining the concequences of circuit-level neural manipulations, enhancing the utility of such emerging technologies^13,29^.

## Methods

### Animals

Sixteen inbred strains of mice were obtained from the Jackson Laboratory, selected to include those in common use and others to enhance genetic diversity: C57BL/6 J, BALB/cByJ, A/J, 129S1/SvImJ, DBA/2 J, C3H/HeJ, AKR/J, SWR/J, SJL/J, FVB/NJ, WSB/Ei, CZECHII/Ei, CAST/Ei, JF1/Ms, MOLF/Ei, and SPRET/Ei. Animals were housed under a standard 24 hour light/dark cycle, consisting of a 12 h day (150 lux overhead illumination) and a 12 h night. Room temperature was 20−22 °C, and mice had *ad libitum* access to water and standard chow (PicoLab Mouse Diet 20, Purina Mills, Richmond, IN). Animals were acclimated to these vivarium conditions for at least 7 days prior to behavioral monitoring. Male mice approximately 3 months of age were examined, with group sizes ranging from *n* = 9 to *n* = 12 per strain. Behavioral data were collected during 7 separate HCM system runs conducted over 11 months, and mice of each genotype were widely distributed among the runs. Experiments were performed in accordance with guidelines of the UCSF Laboratory Animal Resource Center and with the approval of the UCSF Institutional Animal Care and Use Committee.

### Data Collection

Mice were individually housed and monitored for 16 days in HCM cages, each consisting of a Plexiglass enclosure (l/w/h: 45 × 24 × 17 cm) with food and water provided by a feeding monitor and lickometer mounted at one end^5^. An opaque black plastic housing niche (l/w/h: 10 × 10 × 8.7 cm) was located at the opposite end, with a 4 × 4 cm opening at the niche corner closest to the cage center. Cages contained standard UCSF transgenic mouse facility paper bedding, and a cotton nestlet was placed at housing niche opening. Each cage was placed atop an activity platform containing 2 load beam force transducers whose integrated activity enabled the location of the animal’s center of mass to be determined at a rate of 50 measurements per second. A more detailed description of HCM system components had been previously reported^5^.

In each monitoring session, data were collected continuously across days except for a daily maintenance period (Zeitgeber hours 6–8), during which food and water were measured/replaced and nest location noted. Maintenance was performed in a manner that did not require opening of cages, minimizing behavioral disruption. A four day acclimation period to HCM housing was provided, and the data collected during the subsequent 12 days were used for analysis. Quality control algorithms were run to correct activity platform location drift error, and occasional instances of device malfunction were also identified, with data collected during malfunctions excluded from subsequent analysis.

Data were collected over 7 separate monitoring sessions, from 7 independent cohorts of animals over a period of 11 months. Individuals comprising each cohort were genetically heterogeneous; animals of each strain were widely dispersed throughout the sessions. A total of 170 animals were monitored in this study. Within-strain analysis of variance of data collected across testing sessions did not reveal significant effects of cohort as a covariate.

The resulting data provided a record of the spontaneous activity patterns of 170 mice over a total of 1921 Mouse-days (MDs), each of which starts at a maintenance period’s end and runs until the beginning of the next. Formally, each MD of data used as input for all analyses consisted of a series of “Events” of three types. Feeding and drinking events, which numbered in thousands per day, were specified by a time interval. Movement events, which numbered in the tens of thousands per day, were described by a location and time stamp when the distance from the prior recorded location exceeded 1 cm. The amount of chow consumed during each feeding event in a MD was defined as the proportion of total MD feeding time in the event multiplied by the total food consumed in the MD; amounts consumed per drinking event were calculated similarly. Amounts consumed per event were previously^5^ found to be insensitive to time-of-day.

### Spatial Organization

To determine how animals spatially allocate their time, we discretized the cage area into a 12 × 24 array of cells and determined average daily occupancy times for a period of 12 days following an acclimation period. Position density plots were constructed by computing the proportion of time spent within each of the 288 cells. To determine whether animals establish a “Home Base”; i.e. a favored location for periods of inactivity, HCM cages were spatially discretized into a 2 × 4 array of cells (each measuring 11.2 × 12 cm), and occupancy times for each MD were computed as the proportion of time spent at each of the 8 cells. For 158/170 mice, the largest occupancy times occurred in the niche area, which was considered to be their Home Base location. For MDs in which largest occupancy times occurred outside the niche, the cell with occupancy greater than half the total time was designated as the Home Base. For those MDs in which cells with largest occupancy were less than half the total time, the Home Base was designated as the two spatially contiguous cells with highest occupancy times.

### State Designation

The behavioral record was classified into 2 mutually exclusive categories, Active States (ASs) and Inactive States (ISs). To designate ISs, we examined all time intervals occurring between movement, feeding, and drinking events while the animal was outside the Home Base. Those time intervals exceeding an IS Threshold (IST) duration value were classified as ISs; the set of ASs was then defined as the complement of these ISs. Equivalent mathematically, ASs can also be defined as those intervals resulting from connecting gaps between events outside the Home Base of length at most IST; ISs are then defined as the complement of these ASs.

Since the IST is a key factor for AS designation (Fig. S1), we examined its impact on AS properties. For a wide range of ISTs, we calculated: 1) numbers of resulting designated ASs per MD, 2) average AS heritability scores, and 3) the extent to which the resulting AS features enabled discrimination among all strains. We found that a 20 min IST was in the optimal range for heritability and classification, and that AS designation was robust, as indicated by similarities in AS numbers arising from ISTs ranging from 15 to 30 min (Fig. S2). AS pauses briefer than 20 min likely correspond to behaviors that can occur with minimal changes in location (e.g. digging, sniffing, nest building, etc.). All subsequent analyses were performed using a 20 min IST value.

### AS Probabilities, AS Numbers, Food, Water, and Distance

The following quantities were computed for 11 2 h time bins across the day, accounting for a 22 h observation period (excluding the 2 h system maintenance period): 1) Food consumed (F), 2) Water consumed (W), 3) Distance Traveled (D), 4) “AS Probability”, 5) “AS Numbers”, 6) “AS Durations”, and 7–9) “AS Intensities” (for feeding, drinking, and distance). Food/water consumed in a bin are defined as the food/water totals in the intersection of food/water events with the bin, Distance Traveled is the Euclidian distance traversed by the center of mass of the mouse in the time bin, AS Probabilities (ASP) indicate the proportion of bin time spent in ASs, AS Numbers (ASN) are the number of AS intervals in each bin, AS Durations (ASD) are their average duration, and AS Intensities (ASI) indicate the amounts of consumption or Distance Traveled per minute of AS time. Correlations (and their statistical significances) among these features (Fig. S3) were determined using the Pearson and Spearman measures (computed using SciPy routines “stats.pearsonr”, “stats.spearmanr”).

### Heritability Estimates

We quantified the feature variability attributable to genetic vs. environmental factors using a standard linear “analysis of variance” approach^30^ for both a standard feature, Distance Traveled, and AS Probability. Formally, the “Heritability” *H*^2^ of a behavioral feature is defined as the fraction of total feature variance due to strain (sometimes called the “broad-sense” heritability); i.e., *H*^2^ = *V*_*g*_/(*V*_*g*_ + *V*_*e*_), where *V*_*g*_ and *V*_*e*_ are estimates of the genetic and environmental components of the feature’s variance, respectively. For each of the 11 2 h time bins, along with their average, we computed the heritability of a feature as:

$$\begin{array}{c}heritability\,=\frac{\frac{1}{n}(\frac{n}{s-1}{\sum }_{i=1}^{s}{(\mu -{\mu }_{i})}^{2}-\frac{1}{N-s}{\sum }_{i=1}^{s}{\sum }_{j=1}^{n}{({\mu }_{ij}-{\mu }_{i})}^{2})}{\frac{1}{n}(\frac{n}{s-1}{\sum }_{i=1}^{s}{(\mu -{\mu }_{i})}^{2}-\frac{1}{N-s}{\sum }_{i=1}^{s}{\sum }_{j=1}^{n}{({\mu }_{ij}-{\mu }_{i})}^{2})+\frac{1}{N-s}{\sum }_{i=1}^{s}{\sum }_{j=1}^{n}{({\mu }_{ij}-{\mu }_{i})}^{2}},\end{array}$$

where *s* = 16 is the number of strains, *n* = 9 is the number of mice per strain, *N* = 144 is the number of mice used for this analysis, *μ*_*ij*_ is the feature average over MDs for the *j*-th mouse from strain *i*, *μ*_*i*_ is the *i*-th strain mean, and *μ* is the mean over all mice. To determine robustness of the measure, we computed *H*^2^ using a random half of MDs per mouse as input data (and repeated 20 times; “bootstrapping”). More generally, we applied bootstrapping to several of the analyses below as noted.

### Time Budgets

“Total time budgets” were generated, assessing the allocation of time among various behaviors (IS, and within-AS behaviors). In addition, “AS time budgets” were generated to assess allocations of AS time among feeding, drinking, locomotor, and nonlocomotor movement. Because our activity platforms do not allow us to resolve behaviors such as rearing, grooming, sniffing, and digging, nonlocomotor movements that include these behaviors are combined into the category “Other”. AS time budgets were generated in the same manner as Total time budgets, with the exception that IS times were excluded from analysis. The dark cycle and light cycle components of both Total and AS time budgets were also examined. To determine the extent to which these differed, we computed Kullback-Leibler divergence^31^, which provides a distance measure between two probability distributions that is large when the two can easily be distinguished from one another, and small when they cannot.

### Within-AS Temporal Structure

To examine the temporal regulation of behavior within ASs, we aligned AS onsets and determined how the probability of engaging in feeding and drinking varied with the time elapsed from AS onset. We considered the 15 min period following each AS onset, dividing it into 5 s bins. Binary scoring was used, so that bins containing feeding or drinking events were labeled 1 and those without, 0. Thus, each AS was associated with a string of 180 1′s and 0′s for feeding and for drinking. To determine feeding and drinking probabilities, strings derived for all ASs from each MD were added and then divided by the total number of ASs expressed that day. We then averaged per mouse these vectors derived from its MDs. An analogous procedure was used to examine whether changes in feeding and drinking probabilities were associated with AS offsets. Here, we considered the 15 min period prior to each AS offset. For each strain, the peak feeding and drinking probabilities of its population average were compared with those generated using a null model in which feeding and drinking probabilities were assessed relative to a number of randomly-selected movement events matching the average daily number of ASs. For each peak, a Welch’s t-test was used to determine significant differences with the null models (calculated using SciPy routine “stats.ttest_ind” with “equal_var = False”).

### Pair-wise Clustering using Active State Parameters and Distance Traveled

We devised a machine learning approach for determining the strain-specificity of observed behavioral patterns. As a starting point, we determined for all 120 possible pair-wise comparisons (of the 16 strains) the extent to which they were separable using two different feature classes over the 24 h day: Distance Traveled and AS Probability. For each of 20 trials, we randomly assigned half of each animal’s MDs (960 of the 1921 MDs) as a Train set with the remainder designated as a Test set for cross-validation. For each MD and each feature class, we extracted from the data 11 consecutive 2 h time bin quantities or 11-D vectors. Next, for each of the 120 pairwise strain comparisons, we ran the unsupervised *K*-means algorithm (with *K* = 2)^32^ on the train data to obtain two 11-D Train centroids for each pair of strains, representing the average 11-D vector of MDs in each pairwise discrimination (using the SciPy routine “cluster.vq.kmeans”). We then used these Train centroids to determine a Clustering accuracy score for assigning animals correctly between the two strains. This score ranged from 0.5 to 1, with a random performance indicated by 0.5 and a perfect separation by 1.

To score a classification of 2 groups, let *T* be the true vector of *t* 1 s and 2 s corresponding to group 1 and group 2 and let *L* be the vector of 1 s and 2 s with a classifier’s guess as to the labels for group 1 and group 2. The total score is the average of the two sub scores *S*_in_ (rating how well common class membership was detected) and *S*_out_ (rating how discriminative the classification is), which are both between (inclusive) 0 and 1. To calculate *S*_in_, of those labels in *L* for which *T* has 1 s, let *I*_1_ be the count of the most common group; also, of those labels in *L* for which *T* has 2 s, let *I*_2_ be the count of the most common group. Now, set *S*_in_ = (*I*_1_ + *I*_2_)/*t*. To calculate *S*_out_, of those labels in *T* for which *L* has 1 s, let *O*_1_ be the count of the most common group; also, of those labels in *T* for which *L* has 2 s, let *O*_2_ be the count of the most common group. Then, we set *S*_out_ = (*O*_1_ + *O*_2_)/*t*. To arrive at a single clustering score, we take the average of these two: *S* = (*S*_in_ + *S*_out_)/2.

### Strain Classification

We also implemented a strain classifier that used regularized logistic regression^32^ with a cross-entropy loss and a one-versus-rest scheme to classify MDs and mice by strain using HCM features. For each of 20 trials with a random half of MDs chosen as a Train set, we trained the classifier on a feature set to determine strain designations for the other 961 Test MDs in a trial. In this way, we obtained for each feature class, the cross-validated percentage of MDs that were correctly assigned to one of the 16 strains (the probability of labeling a data point correctly by chance in this setting is 6.25%). We performed this analysis using the 11-D ASP and Distance Traveled feature vectors separately, as well as with concatenation of the nine different 11-D HCM MD feature vectors. The accuracy and robustness of classification are indicated by the means and standard deviations, respectively, of MD classification over the trials. Whereas the above procedure revealed classification accuracies based on single MDs, we also sought to classify individual mice using data averaged over multiple MDs. For each of the 20 trials, the Test data (containing half of each animal’s MDs) were used to generate “mouse-averages”. To classify individual mice, we labeled each Test set mouse-average data point with the strain predicted from the classifier determined by the MD Train data. (Regressions performed using routine “linear_model.LogisticRegression” from Python package “sklearn”.) The above procedure was also repeated using 192 MDs as a Train set, and the remaining 90% of data were classified as described above.

### Principal Components Analysis

To better visualize how high cross-validated classification accuracies can be obtained from a few simple behavioral feature classes, we performed a principal components analysis (PCA)^32^. For each of the 170 animals, we averaged over MDs its 2 h binned Distance Traveled or AS Probability feature vectors, and then we extracted from these vectors their top three principle normalized components (each having unit variance). These components capture 95.5% and 74.3% of the variance for Distance Traveled and AS Probability features, respectively (Fig. S5). These PCA vectors can be considered as the three most prominent feature motifs over the 24 h day, which together can capture most of the information in the original feature vectors. We next projected our (mean-zeroed) 170 mouse-average vectors into the 3-D space of these components (for both Distance Traveled and AS Probability feature sets, separately). By averaging projected variables within strains, we obtained for each strain a mean and standard deviation for a 3-D projected feature vector. From these statistics, we produced 3-D ellipsoids representing each strain, with semi-principal axes corresponding to a single standard deviation from the projected strain mean. Due to high levels of variability in Distance Traveled data for the CZECHII/Ei and CAST/Ei strains, plots excluding (Fig. 6c) and including (Fig. S7) these strains were generated.

Mathematically, let *M* be the matrix with each of its 170 rows an 11-D mouse-average ASP (or Distance Traveled) feature vector from a mouse; we assume that *M* has zero column sums (so that each feature has zero mean over the population). Letting $C=\frac{1}{170}{M}^{T}M$ be the covariance matrix for the data, we can find an orthogonal matrix *V* and a diagonal matrix *D* with positive entries decreasing along the diagonal such that *C* = *VDV*^*T*^ (calculated using Python package Numpy routine “linalg.eig”). Projected features are the first three columns of the new matrix *N* = *MVD*^−1/2^, one mouse per row.

To examine how each strain’s 3-D projections overlapped with those of other strains, we computed an “Overlap number” between pairs of strain ellipsoids for each of the two feature classes Distance Traveled and AS Probability (Fig. S6). Given two ellipsoids *V* and *W*, with *V* having larger volume, the Overlap number was computed as the proportion of *W* that belongs to *V*. To estimate the volumes of ellipsoids, we used Monte Carlo sampling using 10,000 points chosen uniformly at random inside a box containing the ellipsoid. To estimate the variability of the Overlap number, we randomly picked half of the MD data, computed mouse averages and their PCA projections, constructed ellipsoids, estimated overlap numbers for all possible 120 ellipsoid pairs, and then repeated for 20 bootstrapping trials to obtain means and standard deviations of the Overlap numbers between pairs of strains.

## Electronic supplementary material

Supplementary Information
