# Supplementary material for: Active State Organization of Spontaneous Behavioral Patterns
Source: Sci Rep. 2018 Jan 18;8:1064. doi: 10.1038/s41598-017-18276-z (PMC5773533; doi:10.1038/s41598-017-18276-z)
Supplement: Supplementary file 1 — Supplementary Information [file 41598_2017_18276_MOESM1_ESM.pdf]

SUPPLEMENTAL FIGURES

Active State Organization of Spontaneous Behavioral Patterns

Hillar C, Onnis G, Rhea D and *Tecott L

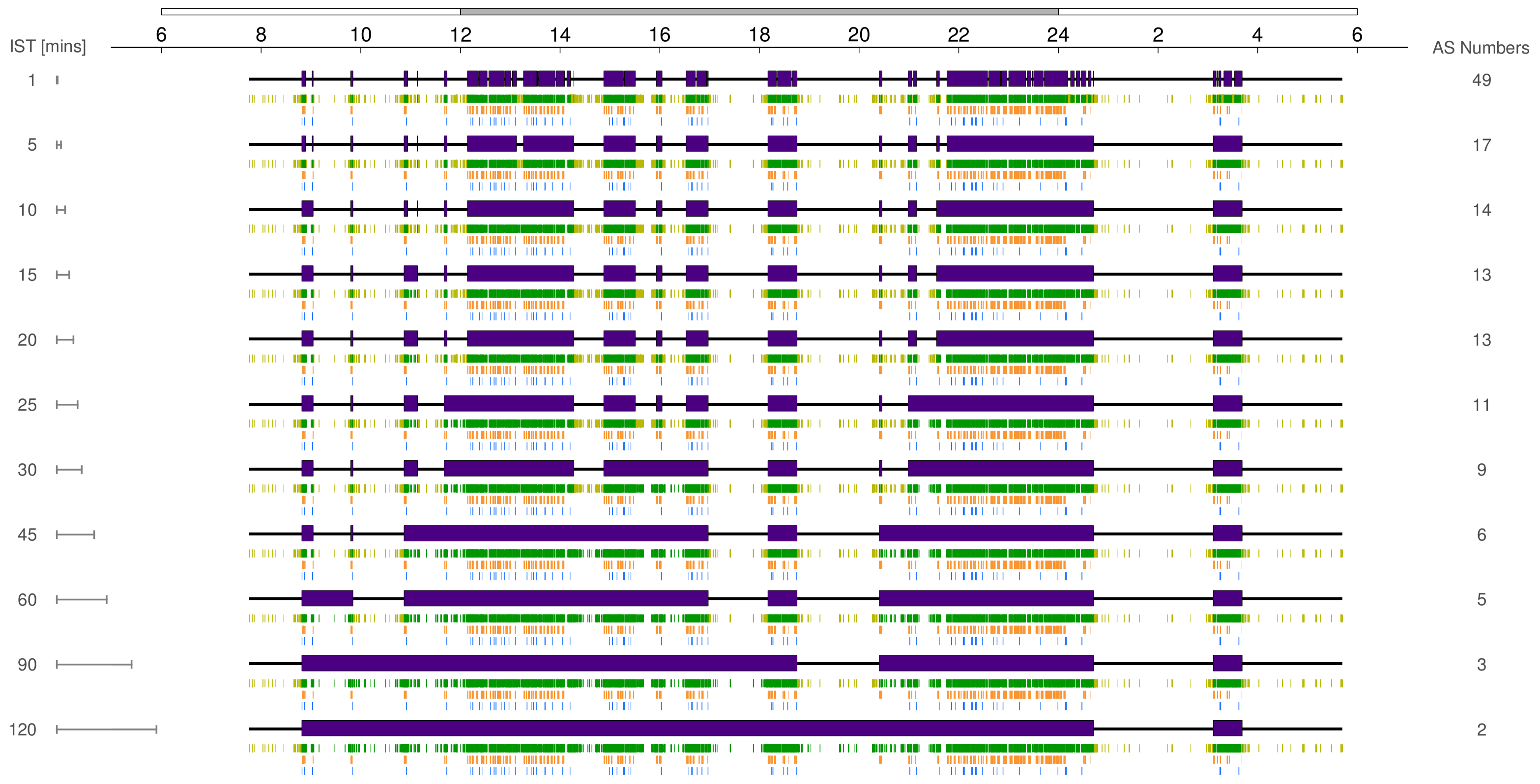

Figure S1: Raster plot representation of the impact of IS Threshold (IST) duration on the designation of ASs. ASs indicated in purple, alternating with ISs (thin black line). Movement events within and outside the niche are shown in yellow and green, respectively. Feeding events are indicated in orange and drinking events in blue. Dark Cycle (ZT 12-24) and Light Cycle (ZT 0-12) are shown by gray and white rectangles above rasters, respectively.

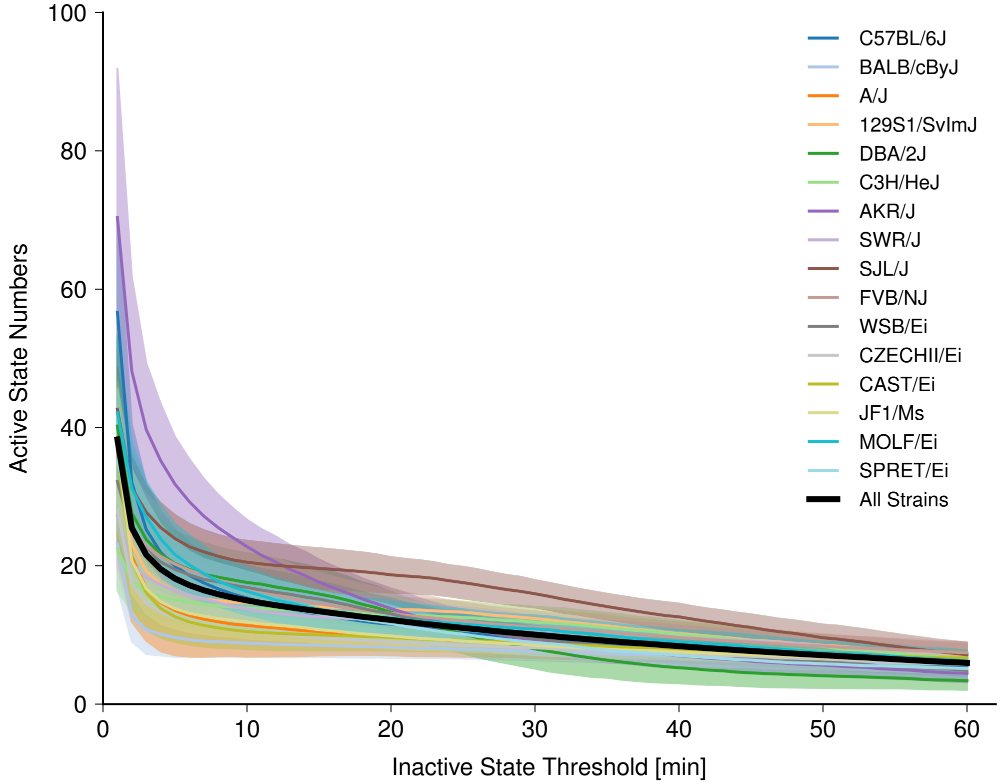

Figure S2: Relationships between IST and resulting number of ASs (mean ± sem) designated for all strains.

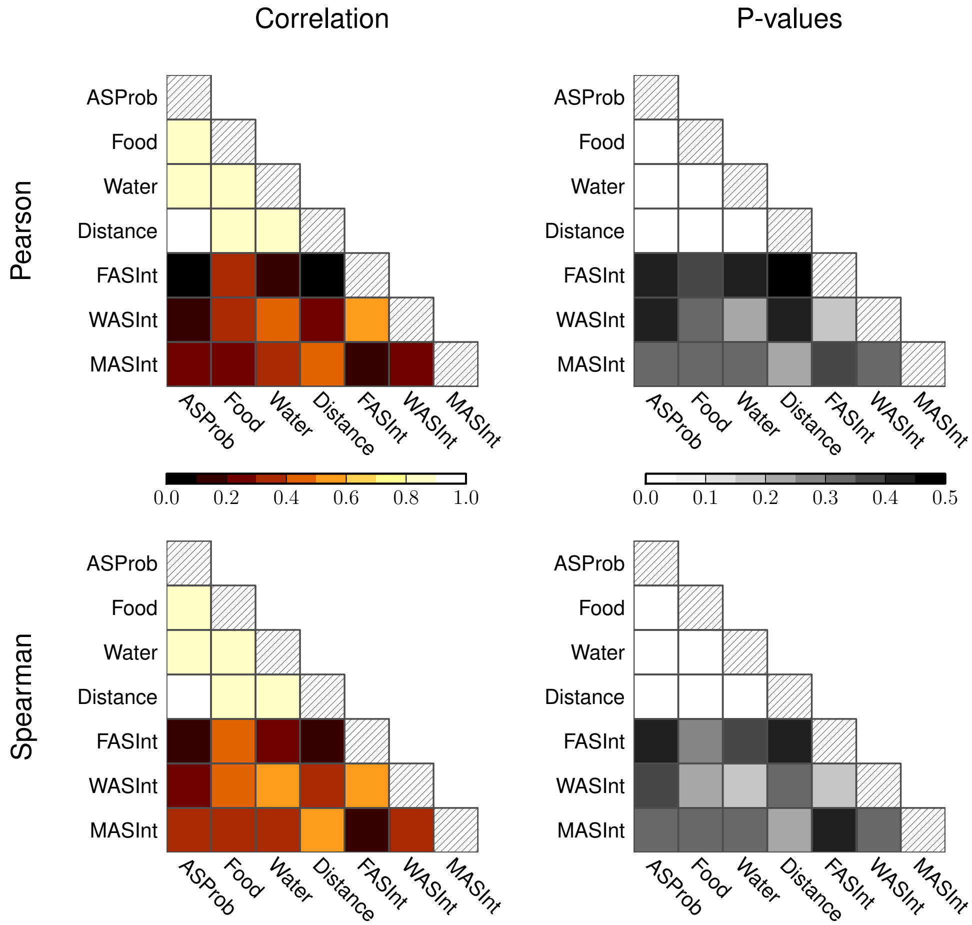

Figure S3: Pearson and Spearman correlation analyses (correlation coefficients and p-values) across all 1921 MDs of the study for food and water intake, Distance Traveled, AS Probability (ASProb), and for AS movement (MASInt), feeding (FASInt), and drinking (DASInt) Intensities.

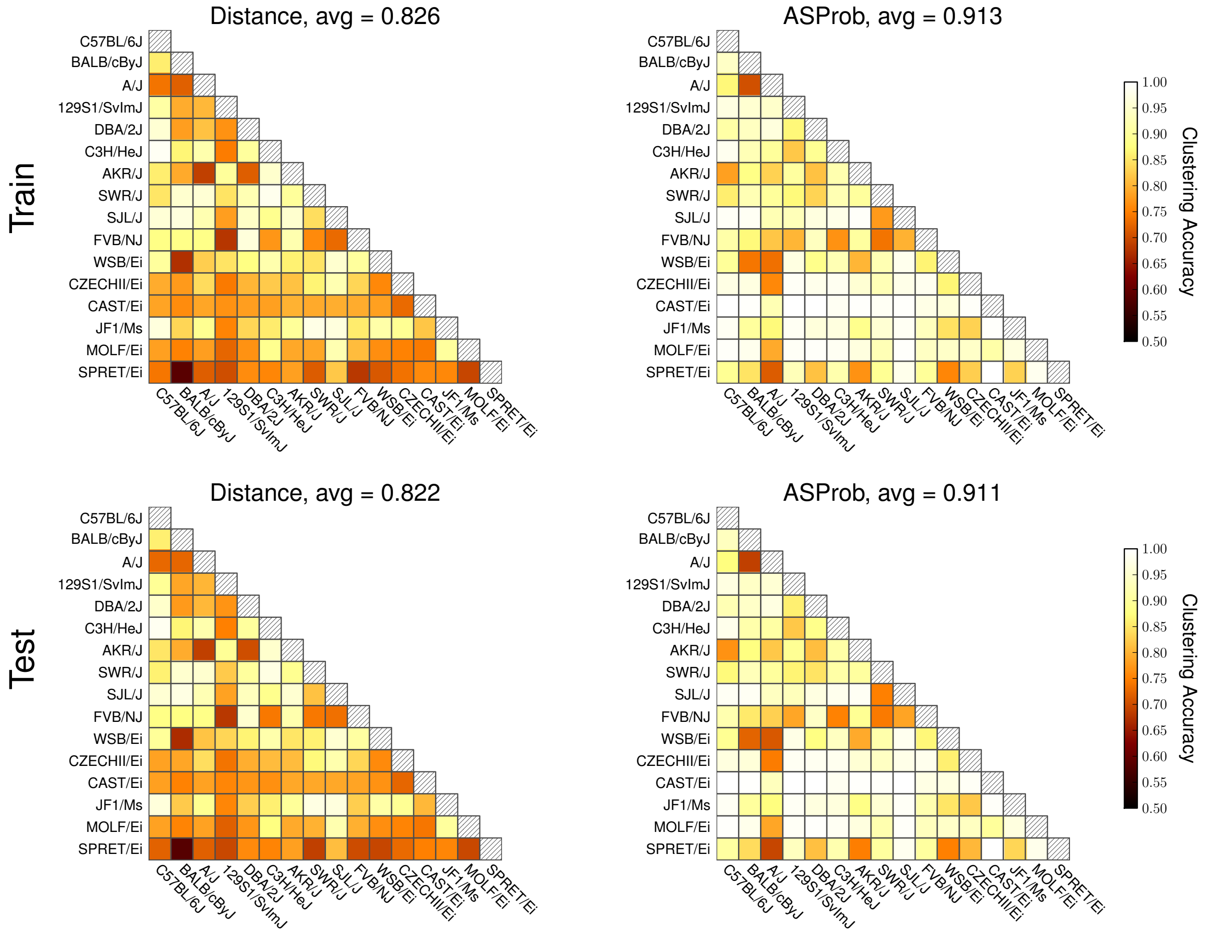

Figure S4: **Pair-wise strain discrimination**. Clustering accuracy scores for each of the 120 possible pairs of strains were determined using either 11-D AS Probability or Distance Traveled feature sets. Clustering accuracy indicated by color coding.

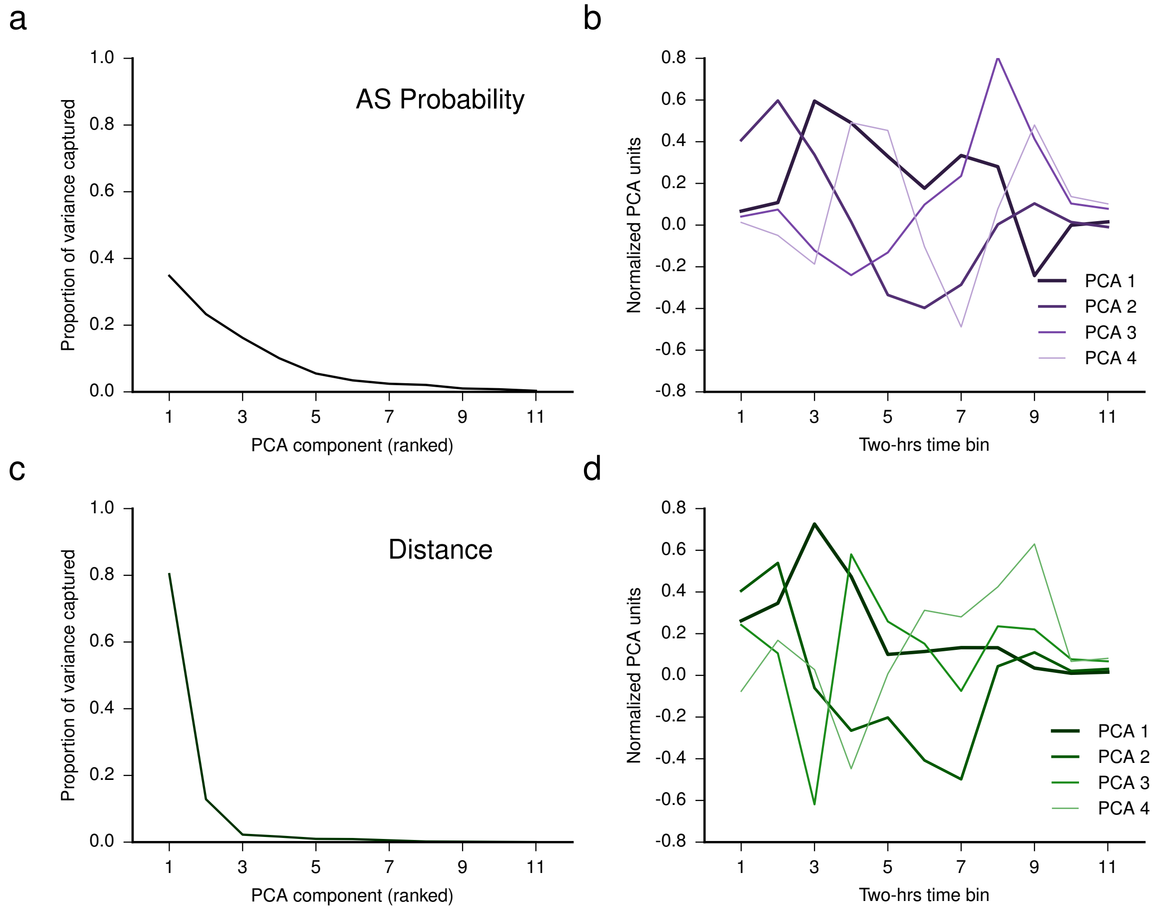

Figure S5: a, c) Proportion of variance captured by principal components of 170 11-D vectors, ordered by magnitude, for AS Probability, resp. Distance traveled, data averaged over MDs for 170 mice in each of 11 2 h time bins. b, d) Top four normalized principal components for data in a), resp. c).

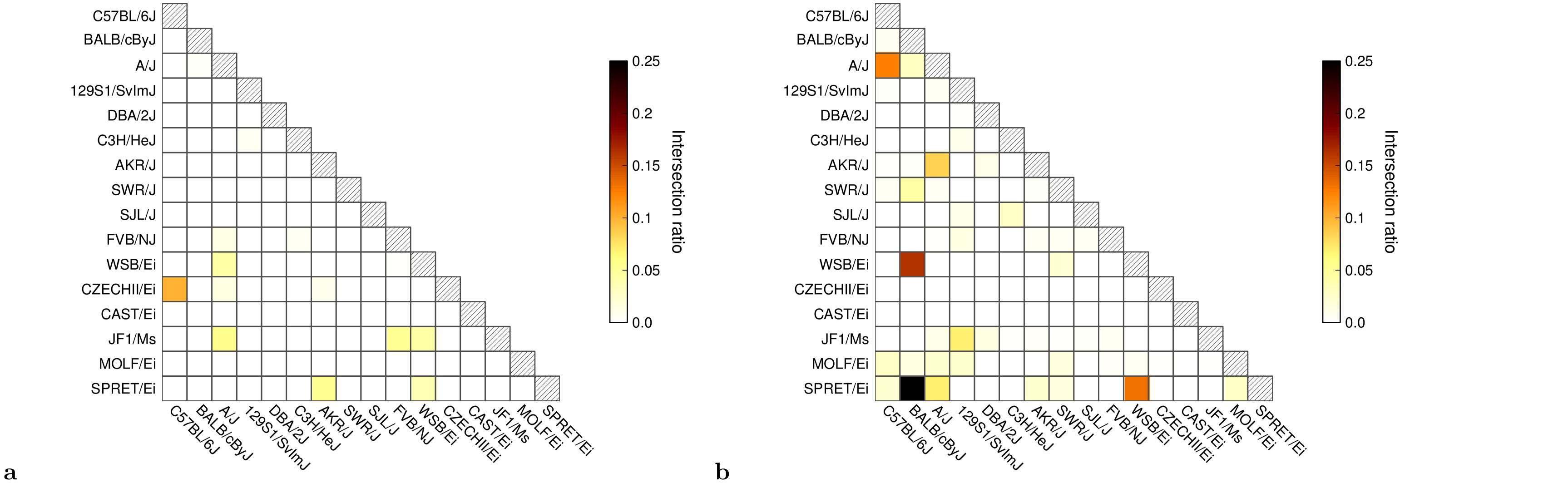

Figure S6: Intersection ratios for each of the 120 possible pair-wise comparisons corresponding to the 3-D ellipsoids, displayed for a) AS Probability and b) Distance Traveled data.
